# Women’s health anxiety and psychological wellbeing during the COVID-19 pandemic. A descriptive study

**DOI:** 10.1590/1516-3180.2021.0155.R1.22042021

**Published:** 2021-08-09

**Authors:** Meltem Akbas, Rukiye Sulu, Ebru Gozuyesil

**Affiliations:** I PhD. Assistant Professor, Department of Midwifery, Faculty of Health Sciences, Cukurova University, Adana, Turkey.; II Midwife and Master’s Student, Department of Midwifery, Health Sciences Institute, Cukurova University, Adana, Turkey.; III PhD. Associate Professor, Department of Midwifery, Faculty of Health Sciences, Cukurova University, Adana, Turkey.

**Keywords:** COVID-19, Women, Pandemics, Anxiety, Psychological wellbeing, Coronavirus disease 2019, Psychological health

## Abstract

**BACKGROUND:**

The rapid spread of the novel coronavirus (COVID-19) outbreak has led to extraordinary measures taken worldwide and has led to serious psychological disorders. With the measures taken, the difficulties in women’s daily lives are increasing exponentially. This situation has caused women to experience more mental health problems.

**OBJECTIVE:**

To identify the relationship between women’s health anxiety and psychological wellbeing and the factors affecting these situations during the COVID-19 pandemic.

**DESIGN AND SETTING:**

Descriptive study conducted online among women living in Adana, Turkey.

**METHOD:**

This descriptive study was conducted among 623 married women between April 1 and April 20, 2020, using a SurveyMonkey online questionnaire. Data were collected using the link that was established. The questions comprised personal information, perceptions regarding the pandemic, the Health Anxiety Inventory (Short Form) and the Psychological Wellbeing Scale.

**RESULTS:**

The women who participated were found to have a high level of anxiety and a moderate level of psychological wellbeing. A positive, moderate-level relationship was found between the scales.

**CONCLUSIONS:**

The COVID-19 pandemic has had negative effects on both physical and psychological health. Support for women, to be provided within their holistic understanding of care, is of great importance for maintaining the psychological health of society.

## INTRODUCTION

The novel coronavirus (COVID-19) emerged in Wuhan, China, in December 2019 and spread all over the world rapidly. It was declared to be a pandemic by the World Health Organization (WHO) on March 11, 2020 (WHO, 2020). When the first case was detected in Turkey, which was on the same date, the pandemic started to affect all parts of society dramatically.^1^^-^^4^ Since then, it has caused many physical, psychological, social and economic changes to people’s lives.^5^ In many countries, including Turkey, people’s practices within daily life have been interrupted by lockdown, social isolation or self-isolation.^6^^-^^8^ Current studies on the COVID-19 pandemic have reported overreactions in society caused by common fear. In particular, it has been reported that individuals who survived and healthcare professionals experienced psychiatric disorders such as anxiety, depression and post-traumatic stress disorder.^9^^-^^12^

Health anxiety is defined as a constant, excessive and irrational worry that is present despite an absence of physical or psychological disease.^13^ Women with high anxiety levels have difficulties in maintaining the activities of their daily lives, through experiencing uneasiness, difficulty in concentration, sleep disorders, fatigue and anger.

Psychological wellbeing is defined as pursuit of a meaningful life through having positive self-perceptions, managing oneself in times of difficulties and identifying strengths and limits for a meaningful life.^14^ Worsening of women’s levels of psychological wellbeing levels could lead to psychological problems, economic losses, exclusion from one’s circles of friends, worsening of family relationships and increased stress levels. COVID-19 causes anxiety because it affects people’s lives negatively and brings many uncertainties to society. Since the virus has a high rate of spreading from person to person, it causes pressure in personal relationships, and the anxiety increases due to uncertainties regarding how long the pandemic will last and how long its effects will continue.^5^^,^^15^ Feelings of anxiety and stress in daily life during the pandemic also have negative effects on psychological wellbeing.^9^^,^^16^

It has been reported in the literature that women are exposed to more stress; they experience psychological problems more commonly; and, compared with men, the prevalence of life-long depression among women is 1.7 to 2.7 times higher.^17^^-^^20^ Traditional patriarchal family structure is dominant in Turkey. For women, meeting their children’s needs, doing housework, cleaning, cooking, etc. and continuing to work from home have increased women’s responsibilities at home during the lockdown. This situation has increased women’s risk of experiencing more psychological problems. Moreover, with the lockdown conditions caused by the pandemic and the social isolation precautions, the economic and social isolation experienced by women has become deeper day by day, and gender-based violence against women has increased incrementally.^12^^,^^21^ Women’s role in maintaining family life is highly important; hence, negative effects on their psychological health are somewhat inevitable in this process. Maintaining women’s health is of great importance in terms of maintaining family life as well as community health.

A review of the literature relating to this topic indicated that numerous studies on people’s psychological health in the COVID-19 pandemic have been conducted.^10^^,^^20^^,^^22^^-^^26^ However, no studies at national level were found to have investigated women’s health anxiety and psychological wellbeing in Turkey during the COVID-19 pandemic.

## OBJECTIVE

The aim of this study was to identify the relationship between health anxiety and psychological wellbeing and the factors affecting this, among women aged between 18 and 60 years during the COVID-19 pandemic.

## METHODS

### Study design and setting

The present research was designed as a descriptive study. The study was conducted among married women living in Adana, Turkey, between April 1 and April 20, 2020.

### Target population and sample

The target population of the study was 435,510 married women aged between 18 and 60 who were living in Adana.^27^ The minimum sample size to represent the female population in this study was calculated using the method of the Australian Bureau of Statistics (with 95% confidence interval and 5% margin of error), which indicated a sample of 535 women.^28^ Considering possible data loss, the sample size was increased by 25%, to become 625 people. During the study period, a total of 865 women living in Adana were approached. However, 142 questionnaires were not included in the analysis because the data had not been filled in accurately. Thus, the study was completed with 623 questionnaires. The study sample comprised volunteer female participants who had the necessary skills for filling in the online form, who were aged between 18 and 60, who were married, who were healthy and who had at least one child.

### Data collection

The questionnaires were put into SurveyMonkey, which is an online questionnaire system (https://tr.surveymonkey.com/r/VGMMZR5). The questionnaire link was shared with women through WhatsApp. The online questionnaire system was set up such that it only allowed one participant per internet protocol address (IP number). Thus, only one questionnaire could be completed by each participant.

### Data collection forms and tools

Data were collected using the link formed by https://tr.surveymonkey.com/r/VGMMZR5. The online data forms contained questions of four types: personal information; perceptions about the pandemic; the Health Anxiety Inventory (HAI) (brief version); and the Psychological Wellbeing Scale (PWS).

### Personal information form

This form was prepared by the researchers in line with the literature and included eight questions about the women’s sociodemographic features.^3^^,^^26^

### Perceptions about the pandemic form

This form was prepared by the researchers in line with the literature^3^^,^^26^ and was composed of nine questions on the women’s perceptions about the COVID-19 pandemic. The participants responded to the questions in this form as “agree” or “disagree”. The questions asked for responses to the following statements “COVID-19 is not as dangerous as it is said to be” (PP1); “COVID-19 is a fatal disease” (PP2); “COVID-19 can infect anyone” (PP3); “COVID-19 can infect men and women with equal probability” (PP4); “COVID-19 vaccination will soon be found” (PP5); “COVID-19 will not infect me if I am careful about my personal hygiene” (PP6); “COVID-19 will not affect me if I am careful about my diet” (PP7); “No matter how many precautions are taken, it might not be possible to prevent COVID-19 infection” (PP8); and “Preventive measures against COVID-19 that are being implemented in Turkey are sufficient” (PP9).

### Health anxiety inventory

The health anxiety inventory (HAI) is an 18-item, self-report scale developed by Salkovskis et al.^29^ Each item is scored between 0 and 3, and the total score ranges from 0 to 54. Higher scores indicate higher health anxiety. The HAI has two subscales, named “Hypersensitivity about Structural and Physical Symptoms” and “Anxiety and Fear of Illness”. The “Hypersensitivity about Structural and Physical Symptoms” subscale is formed by items 1, 2, 3, 4, 5, 6, 7, 8, 9, 10, 11, 12, 13 and 14; and the “Anxiety and Fear of Illness” subscale is formed by items 15,16,17 and 18. The validity and reliability of the HAI for use in Turkey were assessed by Aydemir et al.^13^ The Cronbach’s alpha value of the HAI adapted to Turkish by Aydemir et al. was reported to be 0.918.^13^ In our study, the Cronbach’s alpha value of the HAI was found to be 0.773.

### Psychological wellbeing scale

This scale, developed by Diener et al.^30^ to measure women’s psychological wellbeing, was adapted for use in Turkish by Özmete et al.^31^ The scale consists of three subscales called “General Emotions”, “Satisfaction with Economic, Family and Individual Conditions” and “Maintaining out-of-home activities”. Items 1, 2, 3, 4, 5, 6, 7, 8, 9, 10, 11, 12, 13, 14, 15, 16, 17 and 18 form the General Emotions subscale; items 19, 20, 21, 22, 23, 24, 25, 26, 27, 28, 29, 30, 31 and 32 form the “Satisfaction with Economic, Family and Individual Conditions” subscale; and items 33, 34, 35 and 36 form the “Maintaining out-of-home activities” subscale. The responses to these items are recorded on a 5-point Likert scale as follows: 5 = I totally disagree; 4 = I disagree; 3 = I am not sure; 2 = I agree; and 1 = I totally agree. As some of the items in the scale (items 1, 2, 3, 4, 5, 6, 7, 8, 9, 10, 11, 12, 29, 30, 31, 32, 33 and 34) include negative meanings, these items are scored reversely. Each item is scored between 1 and 5, and the total scores can range from 36 to 180 points. High scores indicate a high level of psychological wellbeing.^31^ The Cronbach’s alpha values of the subscales of the Psychological Wellbeing Scale (PWS) adapted to Turkish by Özmete et al. were reported to be 0.86, 0.88 and 0.86.^31^ In our study, we found the Cronbach’s alpha values of the subscales of the PWS to be 0.75, 0.92 and 0.76.

### Statistical analysis on the data

The data were analyzed using the IBM SPSS statistics software, version 22 (IBM SPSS, Turkey). The normality of the data distribution was assessed using the Shapiro-Wilk test, and the data were found to be distributed normally. The data analysis included descriptive statistical methods (means, standard deviations and frequencies) as well as inter-group assessments of quantitative data, such as through using the independent t test. Data analysis between more than two groups was performed using one-way analysis of variance (ANOVA) test. The groups that caused differences were identified using post-hoc tests. The analysis on relationships between the scales was performed using Pearson’s correlation analysis. The statistical significance level was taken to be P < 0.05.

### Ethical considerations

Academic committee approval was obtained from the Faculty of Health Sciences at our university. A formal document, indicating that no ethics committee approval was needed (since the study was a field study) was obtained from the Medical Faculty Non-Interventional Clinical Research Ethics Committee of our university (Number: 50243401/2020-6; June 2020). In addition, informed consent was obtained from the individuals who participated in the study.

## RESULTS

This study found that 65% of the participating women had a university education or above, 76.7% had children aged 18 and below, 64.7% lived in the city, 62.3% worked and 69.5% perceived that they had a medium-level income (**Table 1**). The average age of the participating women was 38.9 ± 9.87 years (range: 18 to 60); the average number of children aged 18 and younger was 1.29 ± 0.846 (range: 0 to 4); and the average number of children aged 19 and older was 1.00 ± 0.909 (range: 0 to 6).

**Table 1:** Findings regarding comparison of HAI and PWS according to the women’s sociodemographic characteristics (n = 623)

Demographic characteristics			Total HAI		HAI Hypersensitivity to structural and physical symptoms		HAI Fear of anxiety and disease		Total PWS		PWS General emotions		PWS Satisfaction with economic, family and individual conditions		PWS Maintaining out-of-home activities	
	n	%	χ ± SD	Statistical test	χ ± SD	Statistical test	χ ± SD	Statistical test	χ ± SD	Statistical test	χ ± SD1	Statistical test	χ ± SD	Statistical test	χ ± SD	Statistical test
Age																
≤ 30	150	24.1	43.83 ± 2.70	F = 0.410 P = 0.000**	37.16 ± 2.56	F = 1.237 P = 0.291	9.79 ± 1.46	F = 0.447 P = 0.640	103.33 ± 13.49	F = 1.261 P = 0.000**	59.05 ± 12.72	F = 1.604 P = 0.202	30.86 ± 10.36	F = 0.071 P = 0.993	12.21 ± 3.24	F = 0.214 P = 0.808
31-40	228	36.6	44.09 ± 2.75		37.56 ± 2.64		9.64 ± 1.48		102.11 ± 15.66		58.40 ± 12.90		30.59 ± 9.00		12.24 ± 3.48	
≥ 41	245	39.3	44.02 ± 2.97		37.53 ± 2.59		9.67 ± 1.46		101.23 ± 14.61		60.39 ± 11.30		30.89 ± 9.085		12.05 ± 3.13	
Education																
Primary/secondary school	75	12	44.08 ± 2.37	F = 2.035 P = 0.000**	37.94 ± 3.19	F = 1.448 P = 0.228	9.84 ± 1.17	F = 1.097 P = 0.350	103.41 ± 17.00	F = 0.707 P = 0.000**	56.26 ± 14.39	F = 1.296 P = 0.275	31.60 ± 9.83	F = 1.145 P = 0.330	12.18 ± 3.84	F = 0.449 P = 0.718
High school	143	23	44.19 ± 3.83		37.52 ± 2.71		9.58 ± 1.51		101.72 ± 7.87		61.12 ± 10.72		28.52 ± 8.34		12.08 ± 3.16	
University and above	405	65	43.83 ± 2.59		37.30 ± 2.43		9.68 ± 1.40		102.94 ± 12.94		59.32 ± 11.81		31.65 ± 10.36		12.43 ± 3.31	
Working status																
Employed	388	62.3	44.04 ± 2.82	t = 0.478	37.51 ± 2.62	t = 0.760	9.71 ± 1.39	t = 0.621	102.13 ± 13.85	t = -0.316	59.68 ± 12.47	t = 0.880	30.26 ± 8.51	t = -1.779	12.203.26	t = 0.385
Unemployed	235	37.7	43.93 ± 2.83	P = 0.633	37.35 ± 2.58	P = 0.447	9.66 ± 1.58	P = 0.686	102.51 ± 15.451	P = 0.213	58.78 ± 11.92	P = 0.379	31.63 ± 10.58	P = 0.076	12.093.32	P = 0.700
Perceived income level																
High	139	22.3	43.91 ± 2.73	F = 1.642 P = 0.000**	37.54 ± 2.60	F = 0.883 P = 0.435	9.39 ± 1.34	F = 5.670 P = 0.107	100.96 ± 12.03	F = 0.734 P = 0.000**	60.63 ± 11.97	F = 1.607 P = 0.201	28.37 ± 7.94	F = 6.442 P = 0.102	11.963.03	F = 0.870 P = 0.419
Medium	433	69.5	43.95 ± 2.86		37.38 ± 2.61		9.74 ± 1.46		102.67 ± 14.89		59.18 ± 12.95		31.32 ± 9.34		12.163.25	
Low	51	8.2	44.69 ± 2.76		37.84 ± 2.54		10.08 ± 1.69		102.49 ± 16.65		57.18 ± 14.21		32.65 ± 11.81		12.674.17	
Place of residence																
City	403	64.7	44.06 ± 2.68	F = 2.435 P = 0.000**	37.47 ± 2.45	F = 2.475 P = 0.061	9.77 ± 1.47	F = 1.469 P = 0.222	102.45 ± 13.25	F = 0.459 P = 0.000**	59.66 ± 12.24	F = 0.514 P = 0.673	30.63 ± 9.22	F = 0.280 P = 0.840	12.163.30	F = 0.271 P = 0.847
Town	137	22.0	44.27 ± 2.81		37.79 ± 2.70		9.541 ± .42		101.69 ± 16.45		58.28 ± 12.15		31.21 ± 9.59		12.203.30	
Village	83	13.3	43.27 ± 3.35		36.80 ± 3.98		9.57 ± 1.50		102.88 ± 15.90		59.73 ± 12.12		30.96 ± 9.82		12.193.19	

t = independent t test; F = one-way analysis of variance (ANOVA); **P < 0.001

HAI = Health Anxiety Inventory; PWS = Psychological Wellbeing Scale; SD = standard deviation.

**Table 1** also demonstrates the findings relating to comparison of the mean HAI and PWS scores according to the women’s sociodemographic features. Significant differences in total mean HAI scores were detected in relation to the women’s age, education level, income level and place of residence (P < 0.01). Through more detailed analysis, the results indicated that the total mean HAI scores were lower among women who were aged 30 and younger, who had an education level of university and above, whose perceived income was high and who lived in a village (P < 0.05). Significant differences in total PWS scores were found in relation to the variables of age, education level, income level and place of residence (P < 0.01). The more detailed analysis indicated that the total mean PWS scores were higher among women who were aged 30 and younger, who were primary/secondary school graduates, whose perceived income was medium and who lived in a village (P < 0.05).

A statistically significant difference in the women’s total mean HAI scores was found in relation to responses to the question PP2 (P < 0.05). The health anxiety total scores of women who agreed with the statement “COVID-19 is a fatal disease” was found to be lower (**Table 2**). A statistically significant difference in the women’s mean PWS scores was found in relation to responses to the questions PP2 and PP6 (P < 0.05) (**Table 2**). Psychological wellbeing was better among the women who agreed with the statements “COVID-19 is a fatal disease” and “COVID-19 will not infect me if I am careful about my personal hygiene” (**Table 2**).

**Table 2:** Comparison of women’s responses regarding their perception of the pandemic, in relation to the mean scores for HAI and PWS (n = 623)

Questions on perception of the pandemic	n	%	Total HAI		Total PWS	
			χ ± SD	Statistical test	χ ± SD	Statistical test
**PP1**						
Agree	104	16.7	43.73 ± 2.90	t =-1.111	102.13 ± 17.00	P = 0.915
Disagree	519	83.3	44.07 ± 2.80	P = 0.267	102.29 ± 13.94	t = -0.107
**PP2**						
Agree	513	82.3	43.88 ± 2.81	t = -2.265	102.02 ± 15.12	P = 0.032*
Disagree	110	17.7	44.55 ± 2.87	P = 0.025*	103.43 ± 10.80	t =-1.143
**PP3**
Agree	598	96	44.01 ± 2.82	t = 0.291	102.24 ± 14.55	P = 0.819
Disagree	25	4	43.84 ± 3.00	P = 0.771	102.92 ± 12.09	t = -0.271
**PP4**						
Agree	461	74	43.99 ± 2.82	t = -0.185	102.34 ± 2.82	P = 0.830
Disagree	162	26	44.04±2.84	P = 0.853	10.06 ± 13.37	t = 0.214
**PP5**						
Agree	457	73.4	44.04 ± 2.79	t = 0.488	102.06 ± 14.15	P = 0.548
Disagree	166	26.6	43.91 ± 2.92	P = 0.625	102.85 ± 15.28	t = -0.601
**PP6**						
Agree	383	61.5	44.02 ± 2.91	t = 0.157	104.18 ± 12.57	P = 0.009*
Disagree	240	38.5	43.98 ± 2.69	P = 0.876	101.08 ± 15.41	t = -2.619
**PP7**						
Agree	285	45.7	44.03 ± 2.92	t = 0.214	101.42 ± 15.16	P = 0.180
Disagree	338	54.3	43.98 ± 2.75	P = 0.831	102.99 ± 13.82	t = -1.343
**PP8**						
Agree	357	57.3	44.01 ± 2.75	t = 0.098	102.42 ± 13.08	P = 0.766
Disagree	266	42.7	43.99 ± 2.93	P = 0.922	102.07 ± 16.14	t = 0.298
**PP9**						
Agree	268	43	43.86 ± 2.97	t = −1.099	102.73 ± 15.65	P = 0.491
Disagree	355	57	44.11 ± 2.71	P = 0.272	101.92 ± 13.49	t = 0.690

t = independent t test; *P < 0.05.

HAI = Health Anxiety Inventory; PWS = Psychological Wellbeing Scale; SD = standard deviation.

The total mean HAI score of the participating women were found to be 44.00 ± 2.83, and the total mean PWS score was 102.27 ± 14.45 (**Table 3**). The mean scores for other subscales are presented in **Table 3**.

**Table 3:** Mean scores for HAI and PWS among the women (n = 623)

Scales and sub-scales	Min-Max	χ ± SD
**HAI subscales**		
Hypersensitivity to structural and physical symptoms	28-48	37.00 ± 9.00
Fear of anxiety and disease	5-16	8.83 ± 1.78
Total HAI score	34-56	44.00 ± 2.83
**PWS subscales**		
General emotions	18-87	59.34 ± 12.26
Satisfaction with economic, family and individual conditions	14-70	30.77 ± 9.36
Maintaining out-of-home activities	4-20	12.16 ± 12.00
Total PWS score	36-177	102.27 ± 14.45

t = independent t test.

HAI = Health Anxiety Inventory; PWS = Psychological Wellbeing Scale; Min-Max = minimum-maximum; SD = standard deviation.

This study found that there was a positive high-level relationship between the PWS general emotions subscale and HAI total mean scores (P < 0.01). A positive medium-level relationship was detected between the total mean HAI score and the total mean PWS score (P < 0.05). Hence, the level of psychological wellbeing and the level on the general emotions subscale deteriorated as the mean health anxiety scores increased (**Table 4**).

**Table 4:** Relationship between the women’s HAI and PWS scores (n = 623)

Scales and subscales		HAI Hypersensitivity to structural and physical symptoms	HAI Fear of anxiety and disease	HAI Total
PWS	r	0.063	-0.071	0.907
General emotions	P	0.117	0.075	0.000**
PWS	r	0.003	-0.021	0.029
Satisfaction with economic, family and individual conditions	P	0.940	0.597	0.476
PWS	r	0.048	0.002	0.044
Maintaining out-of-home activities	P	0.228	0.956	0.229
PWS	r	0.066	-0.074	0.072
Total	P	0.098	0.066	0.036*

Pearson’s correlation analysis *P < 0.05; **P < 0.001.

HAI = Health Anxiety Inventory; PWS = Psychological Wellbeing Scale.

## DISCUSSION

This study examined the relationship between health anxiety and psychological wellbeing and the factors affecting them, among women during the COVID-19 pandemic.

Comparison of the total mean HAI and PWS scores according to the participating women’s sociodemographic features indicated that the women’s anxiety increased and their psychological wellbeing decreased with increasing age. Tutku et al. reported that women’s health anxiety was significantly higher, and that anxiety levels increased with increasing age.^26^ Oju et al. reported that young age was associated with low anxiety.^24^ They also reported that especially among individuals aged 18 and younger, anxiety scores were low, which was considered to be associated with the facts that morbidity rates were lower among individuals aged younger than 18, they were affected by lockdown less and they were at lower risk of becoming infected. Oju et al. reasoned that since deaths due to COVID-19 were most common among elderly individuals,^24^ higher anxiety and lower psychological wellbeing among these individuals were expected findings in their study. The findings from the present study are in line with those from studies in the literature, in that the levels of anxiety and psychological wellbeing during the pandemic varied depending on age, such that individuals were affected negatively with increasing age.

In the present study, it was found that the anxiety level was lower and psychological wellbeing was moderate among women who had been educated to university and higher levels, i.e. that anxiety levels decreased with increasing education level. Differing from our study, Tutku et al. reported that individuals’ anxiety levels increased with increasing education level.^26^ Qui et al. also reported that individuals who had high education levels experienced high anxiety because they had high awareness about their health conditions.^32^ In comparisons of COVID-19 pandemic management among various countries around the world, Turkey is considered to have managed the process successfully.^3^^,^^33^ This might be a factor relating to the lower health anxiety and positive results regarding psychological wellbeing among women in the high-level education group of the present study, who would be expected to follow the media more closely.

The health anxiety of women living in villages was found to have lower scores in this study. Gao et al. found that anxiety was lower among women living in the countryside than among those living in cities.^34^ People living in cities in Turkey were under lockdown on official holidays and at the weekends in April and May 2020. The city where the present study was conducted was a metropolitan city under lockdown. Hence, the majority of the women were exposed to lockdown, which is considered to increase their anxiety. This would explain the lower anxiety and higher psychological wellbeing of women living in villages.

This study found that women with the perception that they had high income had lower anxiety levels. Erdoğdu et al. investigated individuals’ anxiety levels in Turkey and found that women who had the perception that they had a low level of income had high anxiety levels.^15^ Sümen and Adibelli found that individuals who had low income also had low psychological health levels.^5^ As economic worries were lower among people who perceived that their income was high, low levels of anxiety were an expected result. In line with the literature, this study indicated that these results were somewhat expected during the pandemic period, in which economic problems were triggered.

An analysis on the participating women’s responses about the pandemic demonstrated that 82.3% agreed with the statement “COVID-19 is a fatal disease”, and these women had high health anxiety and poor psychological wellbeing. Disease is a concept that is perceived negatively by people.^35^ The women’s anxiety levels and psychological wellbeing might have been affected negatively because of experiencing restrictive precautions and the individual, social and economic problems caused by them.

Among all the participating women, 61.5% agreed with the statement “COVID-19 will not infect me if I am careful about my personal hygiene”. The psychological wellbeing of the women who agreed with this statement was found to be better than that of women who did not. The importance of personal hygiene in the pandemic process is frequently shown in public service announcements and media, and its importance is highly emphasized. Hence, the women’s psychological wellbeing might have been affected by these factors positively.

The mean HAI score of the women was found to be 44.00 ± 2.83. Considering that the top score is 54, it can be concluded that the pandemic has caused high levels of anxiety among women. In studies on the pandemic process, Erdoğdu et al. and Alan et al. reported that women’s anxiety scores were significantly higher than those of men.^1^^,^^15^ In similar studies conducted during the COVID 19 pandemic period, anxiety levels were found to be high.^22^^,^^23^^,^^25^

The mean PWS score was found to be 102.27 ± 14.45 in our study. According to the assessment criteria for the PWS, the women’s psychological wellbeing was moderate. In previous studies on the pandemic, it was reported to cause deep and tiring negative psychological effects on people. While the pandemic may have caused people without psychological problems to start to have such problems, it may also have worsened the effects on people who already had psychiatric problems.^36^ Wang et al. found that stress, anxiety and depression during the COVID-19 pandemic were highly common, especially among women.^20^ Gao et al. reported that the prevalence of mental health problems was high among their subjects during the COVID-19 pandemic.^32^ Studies have shown that lockdown processes have negative effects on individuals’ psychological wellbeing, particularly women’s.^37^^-^^39^ In a study investigating the effect of gender on psychological wellbeing, Ausin et al. reported that women’s psychological wellbeing levels were significantly lower than those of men.^37^ Fernandez-Abascal and Martin-Diaz reported that women’s psychological wellbeing scores were lower than those of men.^38^ Similar studies on this issue also reported that women had disadvantages in terms of their psychological wellbeing levels.^5^^,^^9^^,^^11^^,^^40^

In the present study, it was found that women’s psychological wellbeing deteriorated as their health anxiety levels increased. Other recent findings have shown that the pandemic and lockdown precautions have increased anxiety levels and have had negative effects on psychological wellbeing. In a study involving 648 students, Sanal Karahan and Hamarta aimed to identify whether solution-oriented thinking had any effects on depression, stress, anxiety and psychological wellbeing. Their regression analysis indicated that solution-oriented thinking had a positive relationship with psychological wellbeing and a negative relationship with depression, anxiety and stress.^40^

### Limitations of this study

This study had some limitations. The scales used in the study were based on the women’s self-reports. Therefore, the responses were based on the women’s subjective perceptions. In addition, since the women’s anxiety and psychological wellbeing levels were not identified before the pandemic, comparisons from before and to after the pandemic were not possible.

## CONCLUSION

The aim of this study was to identify the relationship between health anxiety and psychological wellbeing among women during the COVID-19 pandemic. It was found that anxiety levels were high and psychological wellbeing was medium, among women during the pandemic. Furthermore, women’s health anxiety and psychological wellbeing were found to be affected by several variables. In conclusion, the COVID-19 pandemic has had negative effects not only on physical but also on psychological health. Support provided to women by nurses and midwives within their holistic understanding of care is of great importance for maintenance of the psychological health of society. The results from the present study should be considered for use by policymakers, in formulating interventions to protect, improve and enhance women’s psychological health during this pandemic period. Further studies, including a wider population and larger sample, are recommended.
